# Dimethyl 3,3′-di­meth­oxy­biphenyl-4,4′-di­carboxyl­ate

**DOI:** 10.1107/S1600536814005613

**Published:** 2014-03-15

**Authors:** Fredrik Lundvall, David Stephen Wragg, Pascal D. C. Dietzel, Helmer Fjellvåg

**Affiliations:** a Centre for Materials Science and Nanotechnology, Department of Chemistry, University of Oslo, PO Box 1126, 0315 Oslo, Norway; b inGAP National Centre of Research-based Innovation, Department of Chemistry, University of Oslo, PO Box 1126, 0315 Oslo, Norway; c Department of Chemistry, University of Bergen, PO Box 7803, 5020 Bergen, Norway

## Abstract

In the title compound, C_18_H_18_O_6_, the biphenyl moiety is twisted with a dihedral angle of 29.11 (10)°. The carbometh­oxy groups form C—C—C—O torsion angles of −18.3 (3) and −27.7 (3)° with the attached rings, as a result of steric hindrances from the nearby meth­oxy groups. In the absence of stacking inter­actions and with no H⋯O contacts shorter than 2.7 Å, the packing is dominated by weaker van der Waals inter­actions.

## Related literature   

For the synthesis, see Zhou *et al.* (2007 ▶).
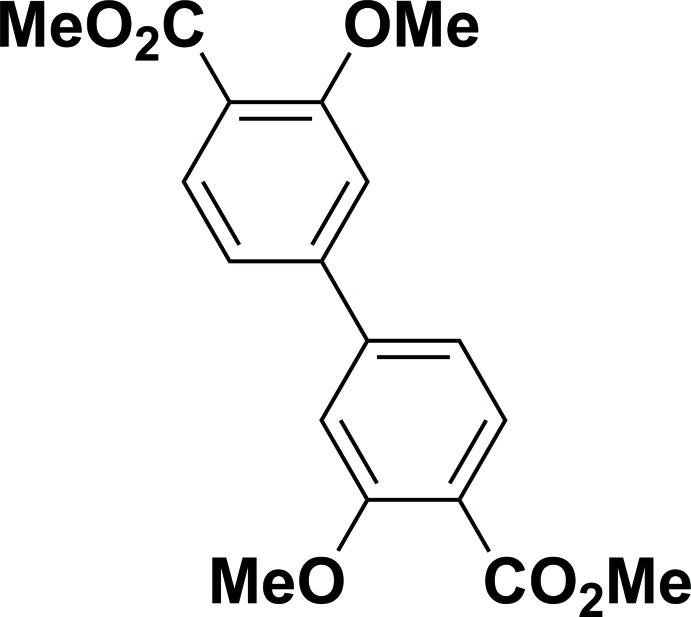


## Experimental   

### 

#### Crystal data  

C_18_H_18_O_6_
*M* *_r_* = 330.32
Monoclinic, 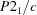

*a* = 12.9320 (6) Å
*b* = 7.3736 (4) Å
*c* = 16.4203 (8) Å
β = 97.410 (2)°
*V* = 1552.69 (13) Å^3^
*Z* = 4
Mo *K*α radiation
μ = 0.11 mm^−1^
*T* = 297 K
0.23 × 0.17 × 0.06 mm

#### Data collection  

Bruker PHOTON CCD diffractometer
Absorption correction: multi-scan (*SADABS*; Sheldrick, 1996 ▶) *T*_min_ = 0.976, *T*_max_ = 0.994
14813 measured reflections
2830 independent reflections
2023 reflections with *I* > 2σ(*I*)
*R*_int_ = 0.031

#### Refinement  

*R*[*F* ^2^ > 2σ(*F* ^2^)] = 0.051
*wR*(*F* ^2^) = 0.143
*S* = 1.02
2830 reflections
217 parameters
H-atom parameters constrained
Δρ_max_ = 0.30 e Å^−3^
Δρ_min_ = −0.21 e Å^−3^

### 

Data collection: *APEX2* (Bruker, 2007 ▶); cell refinement: *SAINT* (Bruker, 2007 ▶); data reduction: *SAINT*; program(s) used to solve structure: *SIR92* (Altomare *et al.*, 1994 ▶); program(s) used to refine structure: *SHELXL97* (Sheldrick, 2008 ▶) and *WinGX* (Farrugia, 2012 ▶); molecular graphics: *DIAMOND* (Brandenburg, 2004 ▶) and *ChemBioDraw Ultra* (CambridgeSoft, 2009 ▶); software used to prepare material for publication: *publCIF* (Westrip, 2010 ▶).

## Supplementary Material

Crystal structure: contains datablock(s) I, New_Global_Publ_Block. DOI: 10.1107/S1600536814005613/ld2122sup1.cif

Structure factors: contains datablock(s) I. DOI: 10.1107/S1600536814005613/ld2122Isup2.hkl

Supporting information file. DOI: 10.1107/S1600536814005613/ld2122Isup3.cml

CCDC reference: 991339

Additional supporting information:  crystallographic information; 3D view; checkCIF report

## Figures and Tables

**Table 1:** Selected torsion angles (°)

C2—C1—C7—C8	28.9 (3)
C3—C4—C13—O1	−27.7 (3)
C11—C10—C14—O4	−18.3 (3)

**Fig. 1.**
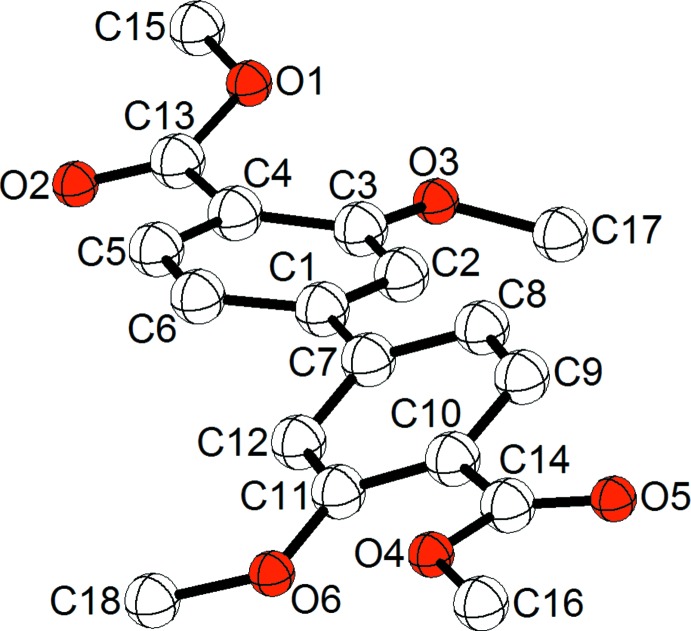
The molecule of the title compound with atom labels and 50% probability displacement ellipsoids. Hydrogen atoms are omitted for clarity.

## supplementary crystallographic information

### 1. Comment

The title compound is an intermediate in the synthesis of 3,3'-dimethoxy-4,4'-biphenyldicarboxylic acid, an organic linker for use in the synthesis of MOFs (Metal-Organic Frameworks). The title compound has previously been reported (Zhou *et al.*, 2007), but its crystal structure was unknown until this publication.

There is a twist between benzene rings, which is a common feature in biphenyl compounds. The methoxy substituents are nearly coplanar with their parent benzene rings. On the opposite, the methyl carboxylate substituents are not co-planar with the adjacent benzene rings, and the corresponding dihedral angles differ between the two halves of the molecule. The methyl groups of the methoxy and methyl carboxylate substituents are oriented away from each other to accommodate the sterical demands of these groups. The long axis of the molecules is oriented in the [101] direction and two-dimensional corrugated layers parallel to the *ac* plane can be imagined. The packing does not appear to be directed by any strong intermolecular bonding, although some long range interaction might influence the ordering of the molecules. Indeed, the carbonyl O atoms O5 and O2 are oriented towards H12 and H2 of neighbouring molecules in a near linear fashion. However, since the O—H distances are very long (>2.7 Å), they are unlikely to be a major factor in the crystal packing.

### 2. Experimental

The title compound was synthesized by a slightly modified version of the method used by Zhou *et al.* (2007). In the Ullmann-coupling of 2 equivalents of methyl 4-iodo-2-methoxybenzoate to form the title compound, the reaction temperature was increased to 225 °C and the reaction time was set to 8 h. The title compound was extracted from the reaction mixture by repeated washing with warm ethyl acetate and subsequent filtering to remove solid particles. The resulting ^1^H NMR spectrum is in good agreement with what was reported by Zhou *et al.* (2007).

Single crystals suitable for XRD analysis were obtained by recrystallizing the title compound from ethyl acetate.

### 3. Refinement

The structure was refined by full-matrix least squares using *SHELXL97* (Sheldrick, 2008) as implemented in the *WinGX* suite (Farrugia, 2012). H-atoms were positioned geometrically at distances of 0.93 (CH) and 0.96 Å (CH_3_) and refined using a riding/rotating model with *U*_iso_ (H)=1.2 *U*_eq_ (CH) and *U*_iso_ (H)=1.5 *U*_eq_ (CH_3_).

### Crystal data

**Table:**

C_18_H_18_O_6_	*F*(000) = 696
*M**_r_* = 330.32	*D*_x_ = 1.413 Mg m^−^^3^
Monoclinic, *P*2_1_/*c*	Mo *K*α radiation, λ = 0.71073 Å
Hall symbol: -P 2ybc	Cell parameters from 5300 reflections
*a* = 12.9320 (6) Å	θ = 2.5–25.3°
*b* = 7.3736 (4) Å	µ = 0.11 mm^−^^1^
*c* = 16.4203 (8) Å	*T* = 297 K
β = 97.410 (2)°	Plate, colourless
*V* = 1552.69 (13) Å^3^	0.23 × 0.17 × 0.06 mm
*Z* = 4	

### Data collection

**Table:**

Bruker PHOTON CCD diffractometer	2830 independent reflections
Radiation source: fine-focus sealed tube	2023 reflections with *I* > 2σ(*I*)
Graphite monochromator	*R*_int_ = 0.031
φ and ω scans	θ_max_ = 25.4°, θ_min_ = 2.5°
Absorption correction: multi-scan (*SADABS*; Sheldrick, 1996)	*h* = −15→15
*T*_min_ = 0.976, *T*_max_ = 0.994	*k* = −8→8
14813 measured reflections	*l* = −19→19

### Refinement

**Table:**

Refinement on *F*^2^	Primary atom site location: structure-invariant direct methods
Least-squares matrix: full	Secondary atom site location: difference Fourier map
*R*[*F*^2^ > 2σ(*F*^2^)] = 0.051	Hydrogen site location: inferred from neighbouring sites
*wR*(*F*^2^) = 0.143	H-atom parameters constrained
*S* = 1.02	*w* = 1/[σ^2^(*F*_o_^2^) + (0.0692*P*)^2^ + 0.5584*P*] where *P* = (*F*_o_^2^ + 2*F*_c_^2^)/3
2830 reflections	(Δ/σ)_max_ = 0.001
217 parameters	Δρ_max_ = 0.30 e Å^−^^3^
0 restraints	Δρ_min_ = −0.21 e Å^−^^3^

### Special details

**Table:**

Geometry. All e.s.d.'s (except the e.s.d. in the dihedral angle between two l.s. planes) are estimated using the full covariance matrix. The cell e.s.d.'s are taken into account individually in the estimation of e.s.d.'s in distances, angles and torsion angles; correlations between e.s.d.'s in cell parameters are only used when they are defined by crystal symmetry. An approximate (isotropic) treatment of cell e.s.d.'s is used for estimating e.s.d.'s involving l.s. planes.
Refinement. Refinement of *F*^2^ against ALL reflections. The weighted *R*-factor *wR* and goodness of fit *S* are based on *F*^2^, conventional *R*-factors *R* are based on *F*, with *F* set to zero for negative *F*^2^. The threshold expression of *F*^2^ > σ(*F*^2^) is used only for calculating *R*-factors(gt) *etc*. and is not relevant to the choice of reflections for refinement. *R*-factors based on *F*^2^ are statistically about twice as large as those based on *F*, and *R*- factors based on ALL data will be even larger.

### Fractional atomic coordinates and isotropic or equivalent isotropic displacement parameters (Å^2^)

**Table:**

	*x*	*y*	*z*	*U*_iso_*/*U*_eq_	
C1	0.65055 (14)	0.1733 (3)	0.91869 (11)	0.0393 (5)	
C2	0.54757 (14)	0.2196 (3)	0.92368 (11)	0.0403 (5)	
H2	0.5274	0.2458	0.9747	0.048*	
C3	0.47394 (14)	0.2277 (3)	0.85443 (11)	0.0381 (5)	
C4	0.50301 (15)	0.1825 (3)	0.77713 (11)	0.0408 (5)	
C5	0.60636 (15)	0.1385 (3)	0.77293 (12)	0.0457 (5)	
H5	0.6270	0.1114	0.7221	0.055*	
C6	0.67954 (15)	0.1336 (3)	0.84176 (12)	0.0466 (5)	
H6	0.7484	0.1038	0.8369	0.056*	
C7	0.72881 (14)	0.1687 (3)	0.99378 (11)	0.0399 (5)	
C8	0.70005 (15)	0.1301 (3)	1.07078 (11)	0.0465 (5)	
H8	0.6307	0.1059	1.0763	0.056*	
C9	0.77472 (15)	0.1280 (3)	1.13896 (11)	0.0457 (5)	
H9	0.7543	0.1020	1.1899	0.055*	
C10	0.87902 (15)	0.1633 (3)	1.13416 (11)	0.0401 (5)	
C11	0.90801 (14)	0.2030 (3)	1.05639 (11)	0.0398 (5)	
C12	0.83282 (14)	0.2061 (3)	0.98785 (11)	0.0404 (5)	
H12	0.8526	0.2338	0.9368	0.048*	
C13	0.43182 (15)	0.1785 (3)	0.69788 (11)	0.0420 (5)	
C14	0.95062 (15)	0.1569 (3)	1.21280 (12)	0.0450 (5)	
C15	0.26093 (16)	0.1350 (4)	0.63245 (12)	0.0577 (6)	
H15A	0.2837	0.0440	0.5969	0.087*	
H15B	0.2584	0.2505	0.6054	0.087*	
H15C	0.1928	0.1045	0.6454	0.087*	
C16	1.12175 (16)	0.1390 (4)	1.27938 (12)	0.0630 (7)	
H16A	1.1179	0.2521	1.3078	0.094*	
H16B	1.1912	0.1218	1.2661	0.094*	
H16C	1.1043	0.0415	1.3139	0.094*	
C17	0.34918 (16)	0.3548 (4)	0.93227 (12)	0.0585 (6)	
H17A	0.3545	0.2607	0.9730	0.088*	
H17B	0.2793	0.4014	0.9245	0.088*	
H17C	0.3969	0.4508	0.9502	0.088*	
C18	1.04057 (16)	0.2687 (4)	0.97237 (12)	0.0604 (7)	
H18A	1.0198	0.1664	0.9380	0.091*	
H18B	1.1149	0.2826	0.9772	0.091*	
H18C	1.0076	0.3763	0.9485	0.091*	
O1	0.33324 (10)	0.1438 (2)	0.70733 (8)	0.0530 (4)	
O2	0.46142 (11)	0.1984 (2)	0.63214 (8)	0.0616 (5)	
O3	0.37416 (9)	0.2830 (2)	0.85684 (7)	0.0475 (4)	
O4	1.04924 (11)	0.1413 (3)	1.20477 (8)	0.0647 (5)	
O5	0.92109 (13)	0.1656 (4)	1.27824 (9)	0.1001 (8)	
O6	1.00999 (10)	0.2403 (2)	1.05180 (8)	0.0579 (5)	

### Atomic displacement parameters (Å^2^)

**Table:**

	*U*^11^	*U*^22^	*U*^33^	*U*^12^	*U*^13^	*U*^23^
C1	0.0377 (10)	0.0468 (11)	0.0326 (10)	−0.0036 (9)	0.0012 (8)	−0.0007 (9)
C2	0.0396 (10)	0.0555 (12)	0.0257 (10)	−0.0045 (9)	0.0039 (8)	−0.0019 (8)
C3	0.0331 (10)	0.0523 (12)	0.0288 (10)	−0.0056 (9)	0.0041 (7)	−0.0010 (8)
C4	0.0408 (11)	0.0524 (12)	0.0291 (10)	−0.0054 (9)	0.0040 (8)	−0.0018 (8)
C5	0.0436 (11)	0.0645 (14)	0.0297 (10)	−0.0037 (10)	0.0073 (8)	−0.0076 (9)
C6	0.0375 (11)	0.0636 (14)	0.0386 (11)	0.0016 (10)	0.0050 (9)	−0.0075 (10)
C7	0.0413 (11)	0.0452 (11)	0.0321 (10)	0.0004 (9)	0.0012 (8)	−0.0003 (8)
C8	0.0382 (11)	0.0651 (14)	0.0361 (11)	−0.0038 (10)	0.0045 (9)	0.0048 (9)
C9	0.0461 (11)	0.0623 (14)	0.0297 (10)	−0.0010 (10)	0.0082 (9)	0.0057 (9)
C10	0.0423 (11)	0.0499 (12)	0.0275 (10)	0.0004 (9)	0.0024 (8)	0.0009 (8)
C11	0.0359 (10)	0.0507 (12)	0.0325 (10)	−0.0005 (9)	0.0033 (8)	0.0007 (8)
C12	0.0408 (11)	0.0537 (12)	0.0265 (10)	−0.0002 (9)	0.0034 (8)	0.0027 (8)
C13	0.0401 (11)	0.0553 (12)	0.0304 (10)	−0.0016 (9)	0.0033 (8)	−0.0034 (9)
C14	0.0458 (12)	0.0606 (13)	0.0285 (10)	−0.0015 (10)	0.0050 (9)	0.0032 (9)
C15	0.0452 (12)	0.0891 (18)	0.0356 (12)	−0.0075 (11)	−0.0071 (9)	−0.0099 (11)
C16	0.0470 (12)	0.107 (2)	0.0326 (11)	0.0060 (13)	−0.0046 (9)	0.0061 (12)
C17	0.0433 (12)	0.0973 (19)	0.0350 (11)	0.0075 (12)	0.0059 (9)	−0.0140 (11)
C18	0.0444 (12)	0.1011 (19)	0.0369 (12)	−0.0048 (12)	0.0101 (9)	0.0112 (12)
O1	0.0411 (8)	0.0887 (12)	0.0277 (7)	−0.0117 (7)	−0.0010 (6)	−0.0036 (7)
O2	0.0497 (9)	0.1084 (14)	0.0272 (8)	−0.0051 (8)	0.0067 (6)	−0.0013 (8)
O3	0.0360 (7)	0.0799 (11)	0.0265 (7)	0.0022 (7)	0.0032 (5)	−0.0055 (6)
O4	0.0444 (9)	0.1215 (15)	0.0266 (8)	0.0118 (9)	−0.0011 (6)	0.0045 (8)
O5	0.0544 (10)	0.217 (2)	0.0287 (9)	0.0005 (12)	0.0049 (7)	0.0034 (11)
O6	0.0368 (8)	0.1072 (13)	0.0289 (7)	−0.0093 (8)	0.0018 (6)	0.0119 (8)

### Geometric parameters (Å, º)

**Table:**

C1—C2	1.387 (3)	C12—H12	0.9300
C1—C6	1.394 (3)	C13—O2	1.200 (2)
C1—C7	1.491 (3)	C13—O1	1.329 (2)
C2—C3	1.387 (3)	C14—O5	1.188 (2)
C2—H2	0.9300	C14—O4	1.304 (2)
C3—O3	1.359 (2)	C15—O1	1.447 (2)
C3—C4	1.410 (2)	C15—H15A	0.9600
C4—C5	1.386 (3)	C15—H15B	0.9600
C4—C13	1.494 (3)	C15—H15C	0.9600
C5—C6	1.378 (3)	C16—O4	1.444 (2)
C5—H5	0.9300	C16—H16A	0.9600
C6—H6	0.9300	C16—H16B	0.9600
C7—C12	1.389 (3)	C16—H16C	0.9600
C7—C8	1.393 (3)	C17—O3	1.422 (2)
C8—C9	1.381 (3)	C17—H17A	0.9600
C8—H8	0.9300	C17—H17B	0.9600
C9—C10	1.386 (3)	C17—H17C	0.9600
C9—H9	0.9300	C18—O6	1.426 (2)
C10—C11	1.407 (2)	C18—H18A	0.9600
C10—C14	1.489 (3)	C18—H18B	0.9600
C11—O6	1.359 (2)	C18—H18C	0.9600
C11—C12	1.389 (3)		
			
C2—C1—C6	118.52 (17)	C11—C12—H12	119.2
C2—C1—C7	120.74 (17)	O2—C13—O1	123.42 (17)
C6—C1—C7	120.74 (17)	O2—C13—C4	123.29 (18)
C3—C2—C1	121.67 (17)	O1—C13—C4	113.25 (16)
C3—C2—H2	119.2	O5—C14—O4	121.94 (18)
C1—C2—H2	119.2	O5—C14—C10	123.11 (19)
O3—C3—C2	122.91 (16)	O4—C14—C10	114.95 (16)
O3—C3—C4	117.51 (16)	O1—C15—H15A	109.5
C2—C3—C4	119.56 (17)	O1—C15—H15B	109.5
C5—C4—C3	118.12 (17)	H15A—C15—H15B	109.5
C5—C4—C13	116.23 (16)	O1—C15—H15C	109.5
C3—C4—C13	125.66 (17)	H15A—C15—H15C	109.5
C6—C5—C4	122.01 (18)	H15B—C15—H15C	109.5
C6—C5—H5	119.0	O4—C16—H16A	109.5
C4—C5—H5	119.0	O4—C16—H16B	109.5
C5—C6—C1	120.08 (18)	H16A—C16—H16B	109.5
C5—C6—H6	120.0	O4—C16—H16C	109.5
C1—C6—H6	120.0	H16A—C16—H16C	109.5
C12—C7—C8	118.60 (17)	H16B—C16—H16C	109.5
C12—C7—C1	119.85 (17)	O3—C17—H17A	109.5
C8—C7—C1	121.54 (18)	O3—C17—H17B	109.5
C9—C8—C7	119.84 (18)	H17A—C17—H17B	109.5
C9—C8—H8	120.1	O3—C17—H17C	109.5
C7—C8—H8	120.1	H17A—C17—H17C	109.5
C8—C9—C10	122.39 (17)	H17B—C17—H17C	109.5
C8—C9—H9	118.8	O6—C18—H18A	109.5
C10—C9—H9	118.8	O6—C18—H18B	109.5
C9—C10—C11	117.76 (17)	H18A—C18—H18B	109.5
C9—C10—C14	116.46 (16)	O6—C18—H18C	109.5
C11—C10—C14	125.78 (17)	H18A—C18—H18C	109.5
O6—C11—C12	122.32 (16)	H18B—C18—H18C	109.5
O6—C11—C10	117.80 (16)	C13—O1—C15	115.73 (15)
C12—C11—C10	119.86 (17)	C3—O3—C17	117.41 (14)
C7—C12—C11	121.53 (17)	C14—O4—C16	116.86 (16)
C7—C12—H12	119.2	C11—O6—C18	117.77 (15)
			
C6—C1—C2—C3	−0.3 (3)	C14—C10—C11—O6	−0.4 (3)
C7—C1—C2—C3	178.88 (18)	C9—C10—C11—C12	0.1 (3)
C1—C2—C3—O3	−175.97 (18)	C14—C10—C11—C12	−179.39 (19)
C1—C2—C3—C4	2.1 (3)	C8—C7—C12—C11	0.9 (3)
O3—C3—C4—C5	175.44 (18)	C1—C7—C12—C11	−179.97 (18)
C2—C3—C4—C5	−2.7 (3)	O6—C11—C12—C7	−179.58 (18)
O3—C3—C4—C13	−4.2 (3)	C10—C11—C12—C7	−0.7 (3)
C2—C3—C4—C13	177.70 (19)	C5—C4—C13—O2	−24.9 (3)
C3—C4—C5—C6	1.6 (3)	C3—C4—C13—O2	154.7 (2)
C13—C4—C5—C6	−178.72 (19)	C5—C4—C13—O1	152.69 (19)
C4—C5—C6—C1	0.1 (3)	C3—C4—C13—O1	−27.7 (3)
C2—C1—C6—C5	−0.8 (3)	C9—C10—C14—O5	−17.9 (3)
C7—C1—C6—C5	−179.99 (19)	C11—C10—C14—O5	161.6 (2)
C2—C1—C7—C12	−150.2 (2)	C9—C10—C14—O4	162.24 (19)
C6—C1—C7—C12	28.9 (3)	C11—C10—C14—O4	−18.3 (3)
C2—C1—C7—C8	28.9 (3)	O2—C13—O1—C15	−1.3 (3)
C6—C1—C7—C8	−152.0 (2)	C4—C13—O1—C15	−178.92 (18)
C12—C7—C8—C9	−0.6 (3)	C2—C3—O3—C17	7.5 (3)
C1—C7—C8—C9	−179.67 (19)	C4—C3—O3—C17	−170.62 (19)
C7—C8—C9—C10	0.0 (3)	O5—C14—O4—C16	−1.3 (4)
C8—C9—C10—C11	0.3 (3)	C10—C14—O4—C16	178.55 (19)
C8—C9—C10—C14	179.8 (2)	C12—C11—O6—C18	−5.2 (3)
C9—C10—C11—O6	179.02 (19)	C10—C11—O6—C18	175.9 (2)

## References

[bb1] Altomare, A., Cascarano, G., Giacovazzo, C., Guagliardi, A., Burla, M. C., Polidori, G. & Camalli, M. (1994). *J. Appl. Cryst.* **27**, 435.

[bb2] Brandenburg, K. (2004). *DIAMOND* Crystal Impact GbR, Bonn, Germany.

[bb3] Bruker (2007). *APEX2* Bruker AXS Inc., Madison, Wisconsin, USA.

[bb4] CambridgeSoft (2009). *ChemBioDraw Ultra* CambridgeSoft Corporation, Cambridge, Massachusetts, USA.

[bb5] Farrugia, L. J. (2012). *J. Appl. Cryst.* **45**, 849–854.

[bb6] Sheldrick, G. M. (1996). *SADABS* University of Göttingen, Germany.

[bb7] Sheldrick, G. M. (2008). *Acta Cryst.* A**64**, 112–122. 10.1107/S0108767307043930 18156677

[bb8] Westrip, S. P. (2010). *J. Appl. Cryst.* **43**, 920–925.

[bb9] Zhou, J., Xu, R.-H., Yang, J., Shen, X., Zhang, J.-J. & Zhu, D.-R. (2007). *J. Nanjing Univ. Tech.* **29**, 16–18.

